# Supplementary material for: Pontoscolex corethrurus: A homeless invasive tropical earthworm?
Source: PLoS One. 2019 Sep 20;14(9):e0222337. doi: 10.1371/journal.pone.0222337 (PMC6754163; doi:10.1371/journal.pone.0222337)
Supplement: S1 Table — (PDF) [file pone.0222337.s001.pdf]

The GLIMMIX Procedure

Model Information	
Data Set	WORK.BIOMASA_PARCIAL
Response Variable	Biomasa
Response Distribution	Gaussian
Link Function	Identity
Variance Function	Default
Variance Matrix Blocked By	Rep
Estimation Technique	Restricted Maximum Likelihood
Degrees of Freedom Method	Containment

Class Level Information		
Class	Levels	Values
Lombriz	2	Bp Pc
Humedad	3	CC Intermed PMP
Alimento	3	Maíz Mucuna Suelo
Rep	5	1 2 3 4 5

Number of Observations Read	1980
Number of Observations Used	1852

Dimensions	
G-side Cov. Parameters	1
R-side Cov. Parameters	1
Columns in X	10
Columns in Z per Subject	1
Subjects (Blocks in V)	5
Max Obs per Subject	382

Optimization Information	
Optimization Technique	Dual Quasi-Newton
Parameters in Optimization	1
Lower Boundaries	1
Upper Boundaries	0
Fixed Effects	Profiled
Residual Variance	Profiled
Starting From	Data

The GLIMMIX Procedure

Iteration History					
**Iteration**	**Restarts**	**Evaluations**	**Objective Function**	**Change**	**Max Gradient**
**0**	**0**	4	25604.038468	.	4.018604
**1**	**0**	4	25604.037879	0.00058877	0.330459
**2**	**0**	2	25604.037875	0.00000384	0.011651

Convergence criterion (GCONV=1E-8) satisfied.

Fit Statistics	
**-2 Res Log Likelihood**	25604.04
**AIC (smaller is better)**	25608.04
**AICC (smaller is better)**	25608.04
**BIC (smaller is better)**	25607.26
**CAIC (smaller is better)**	25609.26
**HQIC (smaller is better)**	25605.94
**Generalized Chi-Square**	1.1133E8
**Gener. Chi-Square / DF**	60339.69

Covariance Parameter Estimates			
**Cov Parm**	**Subject**	**Estimate**	**Standard Error**
**Intercept**	Rep	847.77	713.59
**Residual**		60340	1988.79

Solutions for Fixed Effects								
**Effect**	**Lombriz**	**Humedad**	**Alimento**	**Estimate**	**Standard Error**	**DF**	**t Value**	**Pr > |t|**
**Intercept**				-326.43	21.4225	4	-15.24	0.0001
**Lombriz**	Bp			-47.8962	11.4904	1841	-4.17	<.0001
**Lombriz**	Pc			0	.	.	.	.
**Humedad**		CC		199.90	13.9863	1841	14.29	<.0001
**Humedad**		Intermed		132.46	14.0501	1841	9.43	<.0001
**Humedad**		PMP		0	.	.	.	.
**Alimento**			Maíz	258.42	14.2825	1841	18.09	<.0001
**Alimento**			Mucuna	559.80	14.2906	1841	39.17	<.0001
**Alimento**			Suelo	0	.	.	.	.
**tiempo**				7.0616	0.1816	1841	38.89	<.0001

The GLIMMIX Procedure

Type III Tests of Fixed Effects				
Effect	Num DF	Den DF	F Value	Pr > F
Lombriz	1	1841	17.38	<.0001
Humedad	2	1841	105.35	<.0001
Alimento	2	1841	774.87	<.0001
tiempo	1	1841	1512.50	<.0001

Lombriz Least Squares Means								
Lombriz	Estimate	Standard Error	DF	t Value	Pr > |t|	Alpha	Lower	Upper
Bp	354.73	15.1896	1841	23.35	<.0001	0.05	324.94	384.52
Pc	402.62	15.5036	1841	25.97	<.0001	0.05	372.22	433.03

Differences of Lombriz Least Squares Means Adjustment for Multiple Comparisons: Tukey-Kramer												
Lombriz	_Lombriz	Estimate	Standard Error	DF	t Value	Pr > |t|	Adj P	Alpha	Lower	Upper	Adj Lower	Adj Upper
Bp	Pc	-47.8962	11.4904	1841	-4.17	<.0001	<.0001	0.05	-70.4319	-25.3606	-70.4319	-25.3606

Humedad Least Squares Means								
Humedad	Estimate	Standard Error	DF	t Value	Pr > |t|	Alpha	Lower	Upper
CC	467.79	16.3006	1841	28.70	<.0001	0.05	435.82	499.76
Intermed	400.35	16.3648	1841	24.46	<.0001	0.05	368.25	432.44
PMP	267.89	16.4224	1841	16.31	<.0001	0.05	235.68	300.10

Differences of Humedad Least Squares Means Adjustment for Multiple Comparisons: Tukey-Kramer												
Humedad	_Humedad	Estimate	Standard Error	DF	t Value	Pr > |t|	Adj P	Alpha	Lower	Upper	Adj Lower	Adj Upper
CC	Intermed	67.4446	13.9212	1841	4.84	<.0001	<.0001	0.05	40.1415	94.7476	34.7909	100.10
CC	PMP	199.90	13.9863	1841	14.29	<.0001	<.0001	0.05	172.47	227.33	167.10	232.71
Intermed	PMP	132.46	14.0501	1841	9.43	<.0001	<.0001	0.05	104.90	160.01	99.5019	165.41

Alimento Least Squares Means								
Alimento	Estimate	Standard Error	DF	t Value	Pr > |t|	Alpha	Lower	Upper
Maíz	364.35	16.1922	1841	22.50	<.0001	0.05	332.60	396.11
Mucuna	665.73	16.2048	1841	41.08	<.0001	0.05	633.95	697.51
Suelo	105.94	16.7551	1841	6.32	<.0001	0.05	73.0761	138.80

The GLIMMIX Procedure

Differences of Alimento Least Squares Means
Adjustment for Multiple Comparisons: Tukey-Kramer

Alimento	_Alimento	Estimate	Standard Error	DF	t Value	Pr > |t|	Adj P	Alpha	Lower	Upper	Adj Lower	Adj Upper
Maíz	Mucuna	-301.38	13.6209	1841	-22.13	<.0001	<.0001	0.05	-328.09	-274.67	-333.33	-269.43
Maíz	Suelo	258.42	14.2825	1841	18.09	<.0001	<.0001	0.05	230.40	286.43	224.91	291.92
Mucuna	Suelo	559.80	14.2906	1841	39.17	<.0001	<.0001	0.05	531.77	587.82	526.28	593.32
