# Supplementary material for: Pontoscolex corethrurus: A homeless invasive tropical earthworm?
Source: PLoS One. 2019 Sep 20;14(9):e0222337. doi: 10.1371/journal.pone.0222337 (PMC6754163; doi:10.1371/journal.pone.0222337)
Supplement: S2 Table — (PDF) [file pone.0222337.s002.pdf]

The LOGISTIC Procedure

Model Information	
**Data Set**	WORK.MADUREZ
**Response Variable**	y
**Number of Response Levels**	2
**Model**	binary logit
**Optimization Technique**	Fisher's scoring

**Number of Observations Read**	1980
**Number of Observations Used**	1980

Response Profile		
**Ordered Value**	**y**	**Total Frequency**
**1**	1	598
**2**	0	1382

Probability modeled is y=1.

Class Level Information			
**Class**	**Value**	**Design Variables**	
**lombriz**	**Bp**	1	
	**Pc**	0	
**humedad**	**1**	1	0
	**2**	0	1
	**3**	0	0
**alimento**	**1**	1	0
	**2**	0	1
	**3**	0	0

Model Convergence Status
Quasi-complete separation of data points detected.

Warning: The maximum likelihood estimate may not exist.

Warning: The LOGISTIC procedure continues in spite of the above warning. Results shown are based on the last maximum likelihood iteration. Validity of the model fit is questionable.

The LOGISTIC Procedure

Warning: The validity of the model fit is questionable.

Model Fit Statistics		
Criterion	Intercept Only	Intercept and Covariates
AIC	2427.763	819.932
SC	2433.353	926.158
-2 Log L	2425.763	781.932

Testing Global Null Hypothesis: BETA=0			
Test	Chi-Square	DF	Pr > ChiSq
Likelihood Ratio	1643.8309	18	<.0001
Score	1066.2343	18	<.0001
Wald	326.9750	18	<.0001

Joint Tests			
Effect	DF	Wald Chi-Square	Pr > ChiSq
lombriz	1	1.1977	0.2738
humedad	2	30.6436	<.0001
alimento	2	13.4857	0.0012
tiempo	1	323.0695	<.0001
lombriz*humedad	2	19.2528	<.0001
lombriz*alimento	2	1.8620	0.3942
humedad*alimento	4	3.0342	0.5521
lombri*humeda*alimen	4	15.0633	0.0046

Note: Under full-rank parameterizations, Type 3 effect tests are replaced by joint tests. The joint test for an effect is a test that all the parameters associated with that effect are zero. Such joint tests might not be equivalent to Type 3 effect tests under GLM parameterization.

Analysis of Maximum Likelihood Estimates								
Parameter				DF	Estimate	Standard Error	Wald Chi-Square	Pr > ChiSq
Intercept				1	-5.2234	0.4238	151.9441	<.0001
lombriz	Bp			1	-0.4808	0.4393	1.1977	0.2738
humedad	1			1	2.5103	0.4597	29.8158	<.0001
humedad	2			1	0.8631	0.4404	3.8405	0.0500
alimento	1			1	-17.3064	168.2	0.0106	0.9180
alimento	2			1	-1.6501	0.4495	13.4762	0.0002
tiempo				1	0.0949	0.00528	323.0695	<.0001
lombriz*humedad	Bp	1		1	-0.2032	0.6230	0.1064	0.7443
lombriz*humedad	Bp	2		1	2.3262	0.6339	13.4674	0.0002

The LOGISTIC Procedure

Warning: The validity of the model fit is questionable.

Analysis of Maximum Likelihood Estimates								
Parameter				DF	Estimate	Standard Error	Wald Chi-Square	Pr > ChiSq
lombriz*alimento	Bp	1		1	0.4808	237.8	0.0000	0.9984
lombriz*alimento	Bp	2		1	-0.8669	0.6353	1.8620	0.1724
humedad*alimento	1	1		1	-2.5103	237.8	0.0001	0.9916
humedad*alimento	1	2		1	-0.3806	0.6246	0.3714	0.5423
humedad*alimento	2	1		1	-0.8631	237.8	0.0000	0.9971
humedad*alimento	2	2		1	0.6910	0.6230	1.2300	0.2674
lombri*humeda*alimen	Bp	1	1	1	0.2032	336.3	0.0000	0.9995
lombri*humeda*alimen	Bp	1	2	1	-0.1852	0.8902	0.0433	0.8352
lombri*humeda*alimen	Bp	2	1	1	-2.3262	336.3	0.0000	0.9945
lombri*humeda*alimen	Bp	2	2	1	-3.1366	0.9079	11.9344	0.0006

Odds Ratio Estimates			
Effect	Point Estimate	95% Wald Confidence Limits	
tiempo	1.100	1.088	1.111

Association of Predicted Probabilities and Observed Responses			
Percent Concordant	97.1	Somers' D	0.943
Percent Discordant	2.8	Gamma	0.944
Percent Tied	0.1	Tau-a	0.398
Pairs	826436	c	0.972
