# Supplementary material for: Pontoscolex corethrurus: A homeless invasive tropical earthworm?
Source: PLoS One. 2019 Sep 20;14(9):e0222337. doi: 10.1371/journal.pone.0222337 (PMC6754163; doi:10.1371/journal.pone.0222337)
Supplement: S3 Table — (PDF) [file pone.0222337.s003.pdf]

The GENMOD Procedure

Model Information	
Data Set	WORK.CAPULLOS_TOTAL
Distribution	Zero Inflated Poisson
Link Function	Log
Dependent Variable	capullo

Number of Observations Read	90
Number of Observations Used	90

Class Level Information		
Class	Levels	Values
lombriz	2	Bp Pc
alimento	3	Maiz Mucuna Suelo
humedad	3	CC Intermed PMP

Criteria For Assessing Goodness Of Fit			
Criterion	DF	Value	Value/DF
Deviance		465.3601	
Scaled Deviance		465.3601	
Pearson Chi-Square	80	89.6866	1.1211
Scaled Pearson X2	80	89.6866	1.1211
Log Likelihood		3682.2687	
Full Log Likelihood		-232.6800	
AIC (smaller is better)		485.3601	
AICC (smaller is better)		488.1449	
BIC (smaller is better)		510.3582	

Algorithm converged.

Analysis Of Maximum Likelihood Parameter Estimates								
Parameter		DF	Estimate	Standard Error	Wald 95% Confidence Limits		Wald Chi-Square	Pr > ChiSq
Intercept		1	-22.6553	0.0558	-22.7646	-22.5460	165112	<.0001
lombriz	Bp	1	0.5194	0.0563	0.4090	0.6297	85.15	<.0001
lombriz	Pc	0	0.0000	0.0000	0.0000	0.0000	.	.
alimento	Maiz	1	25.1866	0.0882	25.0137	25.3594	81551.0	<.0001
alimento	Mucuna	0	26.3529	0.0000	26.3529	26.3529	.	.
alimento	Suelo	0	0.0000	0.0000	0.0000	0.0000	.	.

The GENMOD Procedure

Analysis Of Maximum Likelihood Parameter Estimates								
**Parameter**		**DF**	**Estimate**	**Standard Error**	**Wald 95% Confidence Limits**		**Wald Chi-Square**	**Pr > ChiSq**
**humedad**	CC	1	-1.0841	0.0965	-1.2733	-0.8950	126.16	<.0001
**humedad**	Intermed	1	-0.0046	0.0588	-0.1199	0.1107	0.01	0.9374
**humedad**	PMP	0	0.0000	0.0000	0.0000	0.0000	.	.
**Scale**		0	1.0000	0.0000	1.0000	1.0000		

Note: The scale parameter was held fixed.

Analysis Of Maximum Likelihood Zero Inflation Parameter Estimates								
**Parameter**		**DF**	**Estimate**	**Standard Error**	**Wald 95% Confidence Limits**		**Wald Chi-Square**	**Pr > ChiSq**
**Intercept**		1	-0.4498	0.5399	-1.5080	0.6084	0.69	0.4048
**lombriz**	Bp	1	0.4921	0.5738	-0.6325	1.6167	0.74	0.3911
**lombriz**	Pc	0	0.0000	0.0000	0.0000	0.0000	.	.
**humedad**	CC	1	-0.0095	0.6421	-1.2680	1.2490	0.00	0.9882
**humedad**	Intermed	1	-1.5520	0.7759	-3.0727	-0.0314	4.00	0.0455
**humedad**	PMP	0	0.0000	0.0000	0.0000	0.0000	.	.
