# Supplementary material for: Pontoscolex corethrurus: A homeless invasive tropical earthworm?
Source: PLoS One. 2019 Sep 20;14(9):e0222337. doi: 10.1371/journal.pone.0222337 (PMC6754163; doi:10.1371/journal.pone.0222337)
Supplement: Attachment — Submitted filename: Response to Reviewers.docx [file pone.0222337.s004.docx]

Jul 25, 2019

**PlosOne**

**Editor-in-Chief**

**Dear Editor:**

Please find enclosed the manuscript titled (Tracked Changues version) ***Pontoscolex corethrurus:* a Homeless Invasive Tropical Earthworm?** Below is the rebuttal letter in which we list all the changes (point-by-point) we have made to the manuscript.

Thank you again your comments and assistance.

Sincerely yours

Angel I. Ortiz Ceballos

**Rebuttal Letter**

## General remarks

All the corrections indicated referees (*Reviewer # 1 and Editorial Board Editor*) was incorporated on the manuscript. In green, sentences and words eliminated; in red, sentences, words incorporated, and only in the text of the manuscript.

**Specific points:**

***Introduction***

| **Comments:**  Page 4 line 3, 100-120 of the species?  Last sentence page 4, confusing—are you switching back to P. corethrurus here? | ***Page*** | ***Line*** |
| --- | --- | --- |
| **Response:**  *We agree with these comments. We include and delete the following information suggested:*  *Include:*  It has been documented that 3 % of the diversity of earthworms are invasive species (Dupont et al. 2012).  *Delete:*  It has been documented that 3 % (100-120) of the diversity of earthworms are invasive species (Hendrix 2008), and stating that just a few of them had a negative impact on terrestrial agroecosystems would not be an exaggeration (Simberloff 2009; Hendrix et al. 2006) | 3 | 70-71 |

***Materials and Methods***

| **Comments:**  1. In your Methods section, please provide additional location information of the collection sites, including geographic coordinates for the data set if available. 2. In your Methods section, please provide additional information regarding the permits you obtained for the work. Please ensure you have included the full name of the authority that approved the field site access and, if no permits were required, a brief statement explaining why. | ***Page*** | ***Line*** |
| --- | --- | --- |
| **Response:**  *We agree with these comments. We include and delete the following information suggested:*  *Include:*  *Ethics statement*  No permits were required for the collection and laboratory trials. Soil and earthworms were provided by farmers with free of charge.  located near the village Tamulté de las Sabanas (18°08´N, 92°47´W), 30 km east of Villahermosa, Tabasco, Mexico.  at Huimanguillo (79 km southwest Villahermosa, 17°48´N, 93°28´W), | 6  6  6 | 122-124  129-130  138 |

***Results***

| **Comments:**  Page 8, mortality, please state the mortality results more systemaWcally, instead of a few selected treatments. | Page | Line |
| --- | --- | --- |
| **Response**  *We agree with these comments. We include and delete the following information suggested:*  *Include:*  At 100 days of culture, significant effects were observed between the origin of earthworms (OE), food quality (FQ) and soil moisture (SM), and the interaction between these three factors on sexual maturity, number of cocoons, and number and biomass of juveniles (Table 1).  *Mortality*  At the end of the culture, the invasive earthworm (*P. corethrurus*) had a 21.1 % mortality rate in the soil treatment (33 % and 25 % SM), while that of the native earthworm (*B. pearsei*) had only a 1.1 % mortality rate in the soil treatment (only 42 % SM). In the *M. pruriens* and maize stubble treatments (25 %, 33 % and 42 % SM) no mortality was observed in both earthworm species.  *Delete:*  At 100 days of culture, significant effects were observed between the origin of earthworms (OE), food quality (FQ), soil moisture (SM) and mortality, and the interaction between these three factors on sexual maturity, number of cocoons, and number and biomass of juveniles. The invasive earthworm (P. corethrurus) had a 21.1 % mortality rate in the soil treatment (33 % and 25 % SM). In contrast, the native earthworm (B. pearsei) had only a 1.1 % mortality rate in M. pruriens (only 42 % SM). | 8-9 | 181-189 |

**Discussion**

| **Comments:**  Discussion should be wrigen more precisely and directly analysing the results obtained in the experiments performed. | **Page** | **Line** |
| --- | --- | --- |
| **Response:**  *We agree with these comments. We delete the following information suggested:*  *Included:*  This suggets that the four *P. corethrurus* ecotypes described by Taheri et al. (2018a) are likely the result of the selective forces imposed by cultivation, agricultural practices, and industrial and urban activities (Taheri et al. 2018b).  *Eliminated:*  a finding consistent with the absence of P. corethrurus in parcels where maize- and M. pruriens crop rotation is practiced, as well as in pastures and other traditional tropical agroecosystems (Lavelle et al. 1981; Ortiz- Ceballos et al. 2004; Huerta et al. 2006; Marichal et al. 2010; Fragoso et al. 2016; Ortiz-Gamino et al. 2016).  *Included:*  , similar findings have been reported with *P. corethrurus* from fallow (slash-and-burn) to mature forest (Topoliantz and Ponge 2005).  , because it has been suggested that *P. corethrhrus* has a high ability to utilize soil organic resources as an energy source (Lavelle et al. 1987).  *Delete:*  Also, in a Glenrock soil (southern Australia), the survival of the European endogeic earthworm Aporrectodea trapezoides was found in association with soil bacteria (Davidson et al 2013; Menezes et al. 2018).  *Include:*  For example, Topoliantz and Ponge (2005) observed that the behaviour of two populations of *P. corethrurus* separated along the Maroni river (French Guiana, South America) differed significantly: fallow populations produced more cast on charcoal in the presence of forest soil, while the casting activity of the forest population was higher on soil regardless of the soil origin.  *Delete:*  Symbiosis, defined as the interaction between two different organisms living in close physical association, has been acknowledged as a source of evolutionary innovation that allows hosts to exploit otherwise inaccessible niches (Laland et al. 1999; Lund et al. 2010b; Aira et al. 2018). The hologenome (sum of the genetic information of the host and its microorganisms) theory of evolution is based on four generalization: a) all animals and plants establish symbiosis with microorganims; b) microorganisms can be transmitted between generations with fidelity; c) symbiosis affects the fitness of holobionts in their environment; d) genetic variation in holobionts can be enhanced by incorporating different symbiont populations and can change rapidly under enviromental stress (Zilber-Rosenberg and Rosenberg 2008).  *Include:*  Thus, the likely symbiotic evolution of *P. corethrurus* with the microbiome (gut, nephridia and cocoons) should be explored as a source of biogeography and phylogenetic information (Lund et al. 2010a; Brussaard et al. 2012; Davidson et al. 2013; Møller et al. 2015; Zwarycz et al. 2015; Schult et al. 2016; Marchán et al. 2018). That is, we could “…explain why *P. corethrurus* is rare or absent in undisturbed lands” (Lavelle et al. 1987).  *Delete:*  In Eisenia andrei and E. fetida, 275 and 176 bacteria were observed in their cocoons, respectively (Menezes et al. 2018); however, these were dominated by three vertically transmitted (parental) symbionts: Microbacteriaceae, Verminephrobacter, and Ca. Nephrothrix. For example, in a sterile environment, cocoons of M. anomala failed to develop, suggesting a functional relationship between nephridial bacteria of earthworms and soil microbiota (Gilot-Villenave 1994). Besides, it has been noted that during embryogenesis, the nephridial symbiont Verminephrobacter (Betaprototeobacteria) contributes to the biosynthesis of vitamins and the elimination of nitrogen wastes from cocoons, and conserving nitrogen (Schramm et al. 2003; Davidson and Stahl 2006; Lund et al. 2010b; Davidson et al. 2013; Møller et al. 2015).  *Include:*  Until now, only two ecotypes have been recorded (Taheri et al. 2018a) and the criptic linage used in this study corresponds to L1 (the most widespread). The origin of *P. corethrurus* may be related to anthropogenic soil formation (“terras mulatas” and “terras pretas”). The domestication of manioc (bitter and sweet) and peach palm staple food that facilitated sedentary lifestyles in the Amazon region (Lodge 1993; Glaser et al. 2000; Arroyo-Kalin 2010; Clement et al. 2015; Watling et al. 2018; Levis et al. 2018) has evolved to the point that we cannot recognize the predecessors of *P. corethrurus*, as evidenced by the recent designation of the *P. corethrurus* neotype from an anthropogenic environment (James et al. 2019) and temperate climate (Peel 2007), and by the ambiguity used for assigning its place of origin (Righi 1984; Dupont et al. 2012).  *Delete:*  Pyšek et al (2005) observed that archeophyte weeds are common in crops introduced at the beginning of agriculture (cereals), but are poorly represented in crops introduced relatively recently (rape, maize), where neophyte weeds are most numerous.  It is documented that an exchange of domesticated plants (manioc, maize, cacao and others), as well as recently introduced plants (livestock, pastures, coffee, sugar cane and others), took place between the Amazon area and Mesoamerica through agricultural packages that may have also included P. corethrurus as well as weeds, mice, insects, etc. (Denevan 1992; Boivin et al 2016; Levis et al. 208).  *Delete:*  Therefore, the four P. corethrurus ecotypes described by Taheri et al. (2018b) are likely the result of the selective forces imposed by cultivation, agricultural practices, and industrial and urban activities. That is, this species has evolved to develop resistance to herbicides, pesticides, heavy metals, and hydrocarbons, among others (Taheri et al. 2018b). The above may explain why the paradigm of Beddard (1900) " ...tropical earthworms invade only tropical regions (Neotropical) ..." is no longer valid, as P. corethrurus has been recorded across the five biogeographic regions: Nearctic (Gates 1954, Gates 1972, Blakemore 2009), Palearctic (Omodeo et al. 2003; Blakemore et al. 2006; Reynolds and Jones 2006, Sherlock and Carpenter 2009, Blakemore 2009, Cunha et al. 2014, Rota and Jong 2015; Taheri et al. 2018), Ethiopian (Plisko 2001), Indian (Tsai et al. 2000; Blakemore 2009; Singh et al. 2018; Subedi et al. 2018), and Australian (Blakemore 2009). The tropical microclimatic model of Lavelle et al. (1987) for the growth and development of P. corethrurus (20-30 ̊C) is also not applicable to all four P. corethrurus agrotypes (James et al. 2019), since a revision of databases and the literature revealed that this species has been collected in Neotropical latitudes with temperate climate (10- 20 ̊C; 1200 to 2000 m a.s.l.; Köppen 2011; Peel 2007; Volken and Brönnimann 2011) from México (Fragoso 2018; Juárez-Ramón and Fragoso 2014, Ortiz-Gamino et al. 2016), Lesser Antilles (Hendrix et al. 2006), Colombia (Gutiérrez-Sarmiento and Cardona 2014), Brasil (Müller 1857; Bunch et al. 2011; Ferreira et al. 2018; Steffen et al. 2018), Argentina (Mischis and Righi 1999; Mischis 2007), Madagascar (Chapuis-Lardy et al. 2010; Villenave et al. 2010), South África (Plisko 2001, Janion-Scheepers et al. 2016) e India (Singh et al. 2018; Subedi et al. 2018).  The origin of P. corethrurus may be related to anthropogenic soil formation (“terras mulatas” and “terras pretas”) and the domestication of manioc (bitter and sweet) and peach palm staple food that facilitated sedentary lifestyles in the Amazon region (Lodge 1993; Glaser et al. 2000; Arroyo-Kalin 2010; Clement et al. 2015; Watling et al. 2018; Levis et al. 2018), and has evolved to the point that we cannot recognize their wild predecessors, as evidenced by the recent designation of the P. corethrurus neotype from an anthropogenic environment (Müller 1857; James et al. 2019), and by the ambiguity used for assigning its place of origin (Righi 1984; Dupont et al. 2012).  Cryptic invasions occur frequently, often go unnoticed, and are hard to recognize. Also, it is known that cryptic species may display markedly different responses to a given stimulus or stressor (Liebeke et al. 2014; Schult et al. 2016; Morais and Reichard 2018). Therefore, it is important to classify the four P. corethrurus ecotypes (Taheri et al. 2018a; James et al. 2019) as separate evolutionary entities over the residence time, and determine the preference of each ecotype in terms of soil type, culture, and climate, and response to stimulus or stressors (pesticides, herbicides, heavy metals, etc.), among others. To this end, transcriptomes (Rad-Seq) may be used to elucidate the gene flow across cryptic lineages (Puga-Freitas et al. 2015; Schult et al. 2016; Morais and Reichard 2018). In addition, epigenetics could be used to investigate the functional adaptations of P. corethrurus lineages (Kille et al. 2013; Liu et al. 2017; Ponesakki et al. 2017; Fernández-Marchán et al. 2018). Last, potential symbionts may be found for each agrotype, including bacteria, nematodes, enchytraeids and other life forms associated with earthworms (Coates 1990; Fernández-Marchán et al. 2018).  **Comment:**  the conclusion should have a succinct summary about how the evidence from the experiment answers the question posed at the end of the introduction.  **Response:**  *We agree with these comments. We delete the following information suggested*  *Include:*  Based on the results reported here, we conclude that the lower survival and hatching rates of *P. corethrurus* cocoons (offspring) in the agro-ecological niche of *B. pearsei*; it is, a finding consistent with the absence of *P. corethrurus* in parcels where maize- and *M. pruriens* crop rotation is practiced, as well as in pastures and other traditional tropical agroecosystems (Lavelle et al. 1981; Ortiz-Ceballos et al. 2004; Huerta et al. 2006; Marichal et al. 2010; Fragoso et al. 2016; Ortiz-Gamino et al. 2016). This suggests that *P. corethrurus* is an invasive species that thrives far from its natural status, i.e., has no wild ancestry in the study area. Therefore, it is important to determine the preference the four *P. corethrurus* ecotypes (Taheri et al. 2018a) in terms of soil type, cultivation, response to stressors and climate change.  *Delete:*  Based on the results reported here, we conclude that the lower survival and hatching rates of *P. corethrurus* cocoons (offspring) are associated with its symbiotic bacteria, coupled with a higher diversity of the edaphic microbiome in the agro-ecological niche of *B. pearsei.* This suggests that *P. corethrurus* is an exotic species that thrives far from its natural status, i.e., has no wild ancestry in the study area. For this reason, the likely symbiotic eco-evolution of *P. corethrurus* with the microbiome in its gut, nephridia and cocoons should be explored as a source of biogeography and phylogenetic information (Lund et al. 2010a; Brussaard et al. 2012; Davidson et al. 2013; Møller et al. 2015; Zwarycz et al. 2015; Schult et al. 2016; Fernández- Marchán et al. 2018), i.e., going back to the roots (Philippot et al. 2013; Pérez-Jaramillo et al. 2016). | 11  12  12  12  14  14  165 | 255-257  265-266  271-272  277-281  311-316  321-330  331-339 |

***Figures***

*Figures in the text:*

**Comments:**

Page 8-9. Figure 1 does not show growth rates, it shows biomass at 100 days with three food sources. In the second sentence it should say ‘At 100 days the biomass of invasive and naWve species...’

*We agree with these comments. We delete the following information suggested:*

*Include:*

Fig. 1. Biomass of the tropical endogeic earthworms *Pontoscolex corethrurus* (invasive) and *Balanteodrilus pearsei* (native) at 100 days of culture using three diets of different nutritional quality in soil with low anthropic impact. Vertical lines represent standard error.

*Delete:*

Fig. 1. Growth rate of the tropical endogeic earthworms *Pontoscolex corethrurus* (invasive) and *Balanteodrilus pearsei* (native) after 100 days of culture using three diets of different nutritional quality in soil with low anthropic impact. Vertical lines represent standard error.

**Comments:**

Middle of page 10. Fig. 4 does not show cocoon biomass. Page 10-11, there are no Figs 5 and 6 in the manuscript that I have.

*We agree with these comments. We delete the following information suggested:*

*Include:*

Fig. 1. Biomass of the tropical endogeic earthworms *Pontoscolex corethrurus* (invasive) and *Balanteodrilus pearsei* (native) at 100 days of culture using three diets of different nutritional quality in soil with low anthropic impact. Vertical lines represent standard error.

Fig. 2. Sexual maturity (formation of the clitellum) in the tropical endogeic earthworms *Pontoscolex corethrurus* (invasive) and *Balanteodrilus pearsei* (native) at 100 days of culture under the interaction of three diets of different nutritional quality and three moisture content levels in soil with low anthropic impact. Vertical lines represent standard error.

Fig. 3. Number of cocoons produced by the tropical endogeic earthworms *Pontoscolex corethrurus* (invasive) and *Balanteodrilus pearsei* (native) at 100 days of culture under the interaction of three diets of different nutritional quality and three moisture content levels in soil with low anthropic impact. Vertical lines represent standard error.

Fig. 4. Number of juveniles hatched from cocoons produced by the tropical endogeic earthworms *Pontoscolex corethrurus* (invasive) and *Balanteodrilus pearsei* (native) at 100 days of culture under the interaction of three diets of different nutritional quality and three moisture content levels in soil with low anthropic impact. Vertical lines represent standard error.

*Delete:*

We remove figures 4 (cocoon biomass) and 6 (juveniles biomass) from the text; that is, there were many figures.

**References**

| *We included and delete the following references:* | Page | Line |
| --- | --- | --- |
| *Include:*   1. Fragoso, G.C. (2018). Importancia de las lombrices de tierra (Oligochaeta) en el monitoreo de áreas prioritarias de conservación del centro, este y sureste de México. CONABIO. [https://doi.org/10.15468/omvnpi accessed via GBIF.org](https://doi.org/10.15468/omvnpi%20accessed%20via%20GBIF.org) on 2019-05-01 2. Kim, J.S., Sparovek, G., Longo, R.M., de Melo, W.J., & Crowley, D. (2007). Bacterial diversity of terra and pristine forest soil from Western Amazon. Soil Biology & Biochemistry 39: 684-690. 3. Lavelle, P., Maury, M.E., & Serrano, V. (1981). *Estudio cuantitativo de la fauna del suelo en la región de Laguna Verde, Veracruz.* Publicaciones Instituto de Ecología (México) 6:75-105. 4. Lima H.N., Lima, Schaefer E.R., Mello J.W.V., Gilkes R.J., & Ker J.C. (2002). Pedogenesis and pre-Colombian land use of “Terra Preta Anthrosols” (“Indian black earth”) of Western Amazonia. Geoderma 110: 1-17. 5. Lodge, D.M. (1993). Biological invasions: lessons for ecology. Trends in Ecology & Evolution 8(4): 133-137 6. Ortiz-Ceballos, A.I., Fragoso, C. (2004). Earthworm populations under tropical maize cultivation: the effect of mulching with Velvetbean. Biol. Fert. Soils 39, 438-445. 7. Schaefer, C.E.G.R., Lima, H.N., Gilkes, R.J., & Mello, J.W.V. (2004). Micromorphology and electron microprobe analysis of phosphorus and potassium forms of an Indian Black Earth (IBE) Anthrosol form Western Amazonia. Australian Journal of Soil Research 24(4): 401-409. 8. Topoliantz, S., & Ponge, J.F. (2005). Charcoal consumption and casting activity by *Pontoscolex corethrurus* (Glossoscolecidae). Applied Soil Ecology 28: 217-224.   *Eliminated:*   1. Beddard, F.E. (2011). *Earthworms and their allies.* *Earthworms and their allies.* https://doi.org/10.5962/bhl.title.17625 2. Chapuis-Lardy, L., Brauman, A., Bernard, L., Pablo, A. L., Toucet, J., Mano, M.J., Weber, L., Brunet, D., Razafimbelo, T., Chotte, J.L., & Blanchart, E. (2010). Effect of the endogeic earthworm *Pontoscolex corethrurus* on the microbial structure and activity related to CO_2_ and N_2_O fluxes from a tropical soil (Madagascar). *Applied Soil Ecology*, *45*(3), 201–208. <https://doi.org/10.1016/j.apsoil.2010.04.006> 3. Coates, K.A. (1990). Redescriptions of *Aspidodrilus* and *Pelmatodrilus*, enchytraeids (Annelida, Oligochaeta) ectocommensal on earthworms. *Canadian Journal of Zoology*, *68*(3), 498–505. https://doi.org/10.1139/z90-073 4. Cunha, L., Montiel, R., Novo, M., Orozco-Terwengel, P., Rodrigues, A., Morgan, A.J., & Kille, P. (2014). Living on a volcano’s edge: Genetic isolation of an extremophile terrestrial metazoan. *Heredity*, *112*(2), 132–142. https://doi.org/10.1038/hdy.2013.84 5. Fragoso, G.C. (2018). Importancia de las lombrices de tierra (Oligochaeta) en el monitoreo de áreas prioritarias de conservación del centro, este y sureste de México. CONABIO. [https://doi.org/10.15468/omvnpi accessed via GBIF.org](https://doi.org/10.15468/omvnpi%20accessed%20via%20GBIF.org) on 2019-05-01 6. Gates, G.E. (1954). Exotic earthworms of the United States. *Bulletin of the Museum of Comparitive Zoology at Harvard College*, *111*(6), 216–258. 7. Gates, G.E. (1972). Burmese earthworms. An introduction to the systematics and biology of Megadrile Oligochaetes with special reference to Southeast Asia. Transactions of the American Philosophical Sciety, 62, 1-326 8. Gutiérrez-Sarmiento, M.C., & Cardona, C.M. (2014). Caracterización ecológica de las lombrices (*Pontoscolex corethrurus*) como bioindicadoras de suelos compactados bajo condiciones de alta humedad del suelo con diferentes coberturas vegetales (Zipacón, Cundinamarca). *Revista Científica*, *2*(19), 41. <https://doi.org/10.14483/23448350.6493> 9. Hendrix, P.F. (2006). Biological invasions belowground-earthworms as invasive species. In *Biological Invasions Belowground: Earthworms as Invasive Species* (pp. 1–4). https://doi.org/10.1007/978-1-4020-5429-7_1 10. Hendrix, P.F., Callaham MacA., Drake, Jr.J.M., Huang, C.-Y., James, S.W., Snyder, B.A., & Zhang, W. (2008). Pandora’s Box Contained Bait: The Global Problem of Introduced Earthworms. *Annual Review of Ecology, Evolution, and Systematics*, *39*(1), 593–613. <https://doi.org/10.1146/annurev.ecolsys.39.110707.173426> 11. Janion-Scheepers, C., Measey, J., Braschler, B., Chown, S.L., Coetzee, L., Colville J.F., Dames, J., Davies, A.B., Davies, S.J., Davies, A.L.V.,…Wilson J.R.U. (2016). Soil biota in a megadiverse country: Current knowledge and future research directions in South Africa. *Pedobiologia*, *59*(3), 129–174. <https://doi.org/10.1016/j.pedobi.2016.03.004> 12. Kille, P., Andre, J., Anderson, C., Ang, H.N., Bruford, M.W., Bundy, J.G., … Spurgeon, D. J. (2013). DNA sequence variation and methylation in an arsenic tolerant earthworm population. *Soil Biology and Biochemistry 57, 524-532*. <https://doi.org/10.1016/j.soilbio.2012.10.014> 13. Köppen, W., Volken, E., & Brönnimann, S. (2011). The thermal zones of the Earth according to the duration of hot, moderate and cold periods and to the impact of heat on the organic world. *Meteorologische Zeitschrift*, *20*(3), 351–360. https://doi.org/10.1127/0941-2948/2011/105 14. Laland, K.N., Odling-Smee, F.J., & Feldman, M.W. (1999). Evolutionary consequences of niche construction and their implications for ecology. *Proceedings of the National Academy of Sciences*, *96(18)*, 10242–10247. https://doi.org/10.1073/pnas.96.18.10242 15. Liebeke, M., Bruford, M.W., Donnelly, R.K., Ebbels, T.M.D., Hao, J., Kille, P., … Bundy, J.G. (2014). Identifying biochemical phenotypic differences between cryptic species. *Biology Letters*, *10*(9), 20140615. <https://doi.org/10.1098/rsbl.2014.0615> 16. Mischis, C.C. (2007). Catálogo de las lombrices de tierra de la Argentina (Annelida, Oligocheta). En: Brown G.G. & Fragoso C. (eds.), Minhocas: biodiversidade e ecología na America Latina (pp. 241-246). Embrapa So. Londrina, Brasil. 17. Morais, P., & Reichard, M. (2018). Cryptic invasions: A review. *Science of the Total Environment 613, 1438-1448*. <https://doi.org/10.1016/j.scitotenv.2017.06.133> 18. Omodeo, P., Rota, E., & Baha, M. (2005). The megadrile fauna (Annelida: Oligochaeta) of Maghreb: a biogeographical and ecological characterization. *Pedobiologia*, *47*(5–6), 458–465. <https://doi.org/10.1078/0031-4056-00213> 19. Plisko, J. (2001). Notes on the occurrence of the introduced earthworm *Pontoscolex corethrurus* (Müller, 1857) in South Africa (Oligochaeta: Glossoscolecidae ). *African Invertebrates*, *42*(1), 323–334. https://hdl.handle.net/10520/EJC84473 20. Pyšek, P. , Jarošík, V. , Chytrý, M. , Kropáč, Z. , Tichý, L. and Wild, J. (2005). Alien plants in temperate weed communities: Prehistoric and recent invaders occupy different habitats. *Ecology*, *86(3)*, 772–785. https://doi.org/10.1890/04-0012 21. Reynolds, J.W., & Jones, A.G. (2006). The earthworms (Oligochaeta: Acanthodrilidae, Glossoscolecidae, and Lumbricidae) of the Falkland Islands. South Atlantic Ocean*. Megadrilogica* 10(10), 75-86. 22. Rota, E., & de Jong, Y. (2015). Fauna Europaea: Annelida - Terrestrial Oligochaeta (*Enchytraeidae* and *Megadrili*), Aphanoneura and Polychaeta. *Biodiversity Data Journal*, *3*, e5737. <https://doi.org/10.3897/bdj.3.e5737> 23. Sherlock, E., & Carpenter, D. (2009). An updated earthworm list for the British Isles and two new “exotic” species to Britain from Kew Gardens. *European Journal of Soil Biology*, *45(5-6)*, 431–435. https://doi.org/10.1016/j.ejsobi.2009.07.002 24. Singh, S., Singh, J., Sharma, A., Vig, A.P., & Ahmed, S. (2018). First Report of the Earthworm *Pontoscolex corethrurus* (Müller, 1857) from Punjab, India. *International Letters of Natural Sciences*, *68*, 1–8. <https://doi.org/10.18052/www.scipress.com/ilns.68.1> 25. Statsoft. (1999). STATISTICA for Windows (Computer Program Manual). *Statsoft, Inc.* 26. Steffen, G.P.K., Steffen, R.B., Bartz, M.L.C., James, S.W., Jacques, R.J.S., Brown, G.G., & Antoniolli, Z.I. (2018). Earthworm diversity in Rio Grande do Sul, Brazil. *Zootaxa*, *4496*(1), 562–575. https://doi.org/10.11646/zootaxa.4496.1.43 27. Subedi, H.P., Saxena, R.M., & Reynolds, J.W. (2018). New record of an earthworm in the family Glossoscolecidae (Annelida: Oligochaeta) from Sikkim, India. *Megadrilogica*, *23*(2), 51–56. | 17  18  19  19  20  22  23  24 | 386  421  434  439  452  499  534  556 |

***Table***

| **Comments:**  3. Please include your tables as part of your main manuscript and remove the individual files.  **Response:**  *We agree with these comments. We include the following information suggested:*  *Include (page 26, Line 579):* | | | | | | | | | | | | | |  |
| --- | --- | --- | --- | --- | --- | --- | --- | --- | --- | --- | --- | --- | --- | --- |
|  | Table 1. *F*-values and significance levels (ANOVA) of the interaction of three factors on growth and reproduction of the tropical endogeic earthworm *Pontoscolex corethrurus* and *Balanteodrilus pearsei* at 100 days of culture in soil with low anthropic impact | | | | | | | | | | | | | |
|  | **Independent variable** | **Biomass adult** | | **Sexual maturity** | | **Cocoon** | | | | **Juveniles** | | | | |
|  |  |  |  |  |  | **Number** | | **Biomass** | | **Number** | | **Biomass** | | |
|  |  | ***F*** | ***P*** | ***F*** | ***P*** | ***F*** | ***P*** | ***F*** | ***P*** | ***F*** | ***P*** | ***F*** | ***P*** | |
|  | **Origen Earthworm (OE)** | 29.7 | 0.0000 | 22.3 | 0.0000 | 4.4 | 0.0390 | 2238.8 | 0.0000 | 25.1 | 0.0000 | 836.9 | 0.0000 | |
|  | **Soil Moisture (SM)** | 20.4 | 0.0000 | 9.2 | 0.0002 | 22.2 | 0.0000 | 31.7 | 0.0000 | 14.8 | 0.0000 | 35.2 | 0.0000 | |
|  | **Food quality (FQ)** | 191.7 | 0.0000 | 299.4 | 0.0000 | 109.0 | 0.0000 | 12.5 | 0.0004 | 67.0 | 0.0000 | 11.6 | 0.0006 | |
|  | **OE**×**SM** | 0.03 | 0.9739 | 4.2 | 0.0164 | 4.4 | 0.0161 | 9.3 | 0.0000 | 5.0 | 0.0095 | 13.5 | 0.0000 | |
|  | **OE**×**FQ** | 5.2 | 0.0068 | 3.2 | 0.0438 | 11.6 | 0.0000 | 3.5 | 0.0609 | 25.2 | 0.0000 | 10.8 | 0.0010 | |
|  | **FQ**×**SM** | 7.3 | 0.0000 | 2.1 | 0.0897 | 13.4 | 0.0000 | 5.9 | 0.0029 | 11.4 | 0.0000 | 21.7 | 0.0000 | |
|  | **OE**×**FQ**×**SM** | 1.1 | 0.3626 | 2.5 | 0.0442 | 6.4 | 0.0002 | 2.5 | 0.0859 | 6.2 | 0.0002 | 15.3 | 0.0000 | |
