# Supplementary material for: Pontoscolex corethrurus: A homeless invasive tropical earthworm?
Source: PLoS One. 2019 Sep 20;14(9):e0222337. doi: 10.1371/journal.pone.0222337 (PMC6754163; doi:10.1371/journal.pone.0222337)
Supplement: Attachment — Submitted filename: Response to Reviewers R1.docx [file pone.0222337.s005.docx]

August 12, 2019

**PlosOne**

**Editor-in-Chief**

**Dear Editor:**

Please find enclosed the manuscript titled (Tracked Changues version) ***Pontoscolex corethrurus:* a Homeless Invasive Tropical Earthworm?** Below is the rebuttal letter in which we list all the changes (point-by-point) we have made to the manuscript.

Thank you again your comments and assistance.

Sincerely yours

Angel I. Ortiz Ceballos

**Rebuttal Letter**

## General remarks

All the corrections indicated referees (*Editorial Board Editor*) was incorporated on the manuscript. In green, sentences and words eliminated; in red, sentences, words incorporated, and only in the text of the manuscript.

**Specific points:**

***Discussion***

| **Comments:**  …However there are some minor changes which further need to be incorporated into the text before manuscript can be accepted for the publication | ***Page*** | ***Line*** |
| --- | --- | --- |
| **Response:**  *We agree with these comments. We include and delete the following information suggested:*  Delete (Line 261 in revised manuscript spelling of which):  , wich differ from those in the adjacent forest  *Delete* (Line 302 Year of reference 199)*:*  (Daane et al. 199)  *Include:*  (Daane et al. 1999)  *Delete* (Line 332 sentence incomplete changes incorporated in text)*:*  Based on the results reported here, we conclude that the lower survival and hatching rates of *P. corethrurus* cocoons (offspring) in the agro-ecological niche of *B. pearsei* is…  *Include:*  Based on the results reported here, we conclude that the invasive tropical earthworm *P. corethrurus* had lower survival and cocoons hatching rates (offspring) in the agro-ecological niche of the native endogeic earthworm, i.e., a finding…  *Include:*  Acknowledgments  We thank Mario M. Osorio-Arce, Angel Ramos-Sánchez and Efraín Hernández-Xolocotzi, promoters of agroecology in Mexico. In addition, we are grateful to anomymous reviewers and Diana Pérez-Staples for valuable comments and careful revision of the manuscript. | 12  13  15 | 260  302  331 |
